# Patient reported outcome and quality of life measured by a simple questionnaire in patients with symptomatic benign prostate hyperplasia treated by holmium laser enucleation of the prostate (HoLEP)

**DOI:** 10.3389/fsurg.2024.1358701

**Published:** 2024-02-08

**Authors:** Stephanie Schumacher, David Marghawal, Claus Brunken, Jonas Herzberg

**Affiliations:** ^1^ Department of Urology, Krankenhaus Reinbek St. Adolf-Stift, Reinbek, Germany; ^2^ Department of Surgery, Krankenhaus Reinbek St. Adolf-Stift, Reinbek, Germany

**Keywords:** benign prostatic hyperplasia, endoscopic treatment, HoLEP, holmium laser enucleation of the prostate, quality of life, PROM, EEP

## Abstract

**Introduction:**

Holmium Laser Enucleation of the Prostate (HoLEP) is established as an effective transurethral treatment option for LUTS due to BPH with improved postoperative outcome. The aim of this study was to evaluate the medium-term results by patient reported outcome measurement and to detect potential risk factors for postoperative complications or impaired outcome.

**Methods:**

We performed a retrospective single-center cohort study including all patients undergoing HoLEP in the study center between April 2019 and December 2021. Therefore, perioperative parameters and postoperative outcome was documented and all patients were asked for their outcome (PROM), complications, IPSS, QoL and changes in sexual and continence function by a questionnaire at a single time point.

**Results:**

In the study period, a total of 541 patients with a mean age of 72.5 ± 8.4 years were treated by HoLEP in the study center. 71.7% of the questionnaires were returned after a mean observation period of 14.9 ± 6.3 month. 91% of the patients reported to the single-timepoint questionnaire reporting a good satisfaction with the procedure and a low postoperative complication rate. The international prostate symptom score could be reduced significantly to 6.2 ± 5.7 (preoperative 19.0 ± 7.2; *p* < 0.001). Patients with an ASA score ≥ 3, prostate volume > 80 ml, medication with platelet inhibitors or DOAK or preoperative need of an indwelling catheter didn't show an increased complication rate.

**Conclusion:**

The overall satisfaction with the procedure and its results are high. We could not identify any independent risk factors for postoperative complications after HoLEP. The used questionnaire is a simple tool for postoperative patient reported outcome measurement with a good correlation to clinical parameters.

## 1 Introduction

Age is a significant predictor for development of benign prostate hyperplasia (BPH) and subsequent Lower Urinary Tract Symptoms (LUTS) (1, 2). In the European EPIC study Riboli et al. reported an incidence of storage and voiding LUTS of about 51% and 26% of men evaluated, respectively (3, 4). Numerous studies prove the correlation between the increase in prostate volume and increasing age. An increase in volume of 2% to 2.5% per year of life is considered physiological (5). At the same time, comorbidities, incidence of anticoagulating drugs, the severity of LUTS and the need for treatment of BPS increase with age (1). Till now, no preventive options are available. If the conservative treatment is (no longer) efficient, surgical options should be evaluated to avoid bothering symptoms, urinary retention and consecutive complications, lowering not only patients' quality of life (QoL) but also the QoL of their partners (6).

The transurethral resection of the prostate (TUR- P) has been considered to be the reference procedure in the interventional therapy. Beside this procedure different laser-assisted techniques have been established which use different techniques of tissue ablation—vaporization, resection or enucleation (7).

As one of these laser-assisted techniques Holmium laser enucleation of the prostate (HoLEP) as an enucleating procedure was investigated in numerous studies and could be confirmed as an effective, safe procedure for the treatment of BPS (8–11). After the first invention by Gilling and Frauendorfer in the late nineties (12), the surgical techniques of HoLEP have been constantly developed and are still developing with progressively better results (11). Following this, HoLEP is recommended by different national guidelines as a size-independent endoscopic treatment option (13, 14). Because of this broad recommendations and high evidence, HoLEP can be named the new gold standard for BPS treatment (15, 16).

One reason for the good outcome is the technique of enucleation. Holmium laser uses a relatively short wavelength which is responsible for the low depth of tissue penetration, but allowing an effective coagulation (17). The pulsed emission of the laser energy leads to very high peak energy levels resulting in a mechanic tissue interaction (chisel effect). This mechanic component supports the tissue dissection in the correct plane. These features support the complete removal of the adenoma analogous to the open simple prostatectomy. The anatomically correct tissue preparation under direct vision allows a sufficient haemostasis. This results in an advantage for patients under anticoagulation allowing a relatively safe procedure in this subgroup (18–20).

Postoperative outcome after HoLEP is commonly measured by peak urinary flow (*Q*_max_), post void residual (PVR) and the international prostate symptom score (IPSS) (1, 21). Results of a single uroflowmetry can be impaired by patient's tension and circadian rhythms (22). Due to the need for a visit, this standard follow-up is time-consuming. Patient reported outcome measurement (PROM) needs much less effort and is therefore more suitable for the broad use for follow-up and quality control. The Patient Global Impression of Improvement (PGI-I) questionnaire shows a good correlation with changes in IPSS and QoL after surgery for bladder outlet obstruction (23). PROM can assess a number of different health domains, ranging from individual or clusters of symptoms to functional domains and even broader concepts such as general health status and health-related quality of life (HRQoL) (24). PROM are well established for follow-up after surgery for pelvic floor disorders (25), urethroplasty and after radical prostatectomy. In endourology PROM is mainly focused on stone management (26). Apart from this, PROM evaluation the patients' satisfaction and improvement of LUTS is rarely reported (1). The optimal tool for this evaluation and the impact of postoperative complications and risk factors on patients' satisfaction are still under discussion.

The aim of this study was to evaluate risk factors for postoperative complications and impaired outcome after HoLEP and to determine the accuracy of the PROM using the PGI-I in an unselected “real-life” patient cohort in a single center retrospective cohort study.

## 2 Methods and materials

### 2.1 Study cohort and data collection

All patients who underwent HoLEP for the treatment of BPS between April 2019 and December 2021 were included into this retrospective cohort study. There were no exclusion criteria. The indication for enucleation was made in accordance with the national guidelines (13).

Patients were included on the basis of the procedural code for HoLEP. After inclusion, medical records were analyzed to evaluate potential risk factor for postoperative complications such as anticoagulation, large adenoma, urinary retention or high ASA score. Afterwards, a standardized questionnaire was sent out postoperatively at a single timepoint. This questionnaire was separated into different parts which include the IPSS, QoL, the PGI-I (Table 1), enquiry of postoperative complications according to the Dindo-Clavien classification (27), the change in sexual function and urinary continence. For the last two questions used the same graduation like the PGI-I.

**Table 1:** Patient global impression of Improvement (PGI-I) questionnaire.

Check the one number that best describes how your urinary tract condition is now compared with how it was before your operation	
1	Very much better
2	Much better
3	A little better
4	No change
5	A little worse
6	Much worse
7	Very much worse

In addition to this questionnaire, patients' preoperative characteristics were extracted from the medical records. This included age, previous therapy for BPS, urinary retention, preoperative *Q*_max_, PVR and IPSS, as well as ASA status and preoperative medical conditions and medication. Preoperative prostate volume and postoperative histology were additionally extracted. We considered the weight of the resected tissue, the operative time, the postoperative hospital length of stay and the blood transfusion rate as peri- and postoperative parameters.

Transient incontinence after HoLEP is an issue. In more than 80% of the patient's recovery takes less than 3 months (28). It was aimed to evaluate the results after the complete recovery of the patients. Therefore, the questionnaire was sent to the patients no earlier than 6 months after surgery, to assess the results after complete recovery.

An additional postoperative visit of the patient for postoperative assessment was not performed.

### 2.2 Surgical procedure

#### 2.2.1 Preoperative setting

The preoperative work-up included a urine test and, in case of the presences of an infection, a urine culture was collected. Depending on the findings, preoperative antibiotic therapy was given for at least 3 days and continued postoperatively. A transurethral catheter was changed preoperatively. Patients with normal urine findings received only perioperative antibiotic prophylaxis with a second-generation cephalosporin.

A monotherapy with antiplatelet agent was continued throughout the surgical procedure. Anticoagulation agents were stopped 48 h before surgery and a bridging therapy was started depending on indication and patients' characteristics.

#### 2.2.2 Intraoperative setting

All procedures were performed by a single surgeon, who has completed his learning curve prior to this study period. A high-power holmium laser (Quanta CyberHo, 100 watts, Quanta System S.p.A, Milan, Italy) was used for this procedure. All surgical procedures were performed with a laser energy of 81 watts and a pulse duration of 160 μs. The first step was always urethral calibration and an exploration of the urinary bladder. After exclusion of any contraindications, the enucleation was performed using the “two lobe” technique (29). An incision was performed at the 5 o'clock position to reach the surgical capsule. The enucleation started always apical. As soon as the apical adenoma was dissected from the capsule the mucosa at the sphincter region was incised to avoid unnecessary tension to the sphincter region. The lateral lobes were separated ventrally with a 12 o'clock incision. After enucleation of the left lateral lobe the medium and right lateral lobe were removed together. The adenoma was removed from the urinary bladder using a morcellator (Piranha; Richard Wolf GmbH, Knittlingen, Germany). A transurethral 3-way-irrigation catheter with a diameter of 22 charrier was inserted and blocked in the prostate fossa. A continuous irrigation with saline was started and continued throughout the postoperative period.

#### 2.2.3 Postoperative procedure

The transurethral catheter was removed within 48 h after surgery in the absence of bleeding. After initial micturition, residual urinary sonography was performed and the patients were discharged if findings were inconspicuous. In case of larger amounts of residual urine, an individual decision was made on the insertion of a new transurethral catheter.

If no persisting bleeding was observed, anticoagulation therapy was started again 48 h after surgery.

### 2.3 Ethical approval

Ethical approval was waived by the Ethics Committee of the Medical Association Schleswig-Holstein due to data protection of the data collected by the retrospective non-interventional study design. Additional written informed consent was not needed. The study has been submitted and registered in the German Register of Clinical Trials (DRKS00024746).

### 2.4 Statistical analysis

Statistical analysis was performed using SPSS version 29 (IBM, Armonk, NY, USA). Univariate analyses were performed using *t*-test and Mann–Whitney-*U*-test for continuous variables. Chi-Square test or Fisher's exact test was used for categorial data. Results are given in mean with standard deviation for continuous variables and as percentage for categorical variables. Comparing two groups before and after surgery, Wilcoxon test was used. All reported *p*-values were considered statistically significant if *p* < 0.05.

The data presented in this study are reported in concordance with the STrengthening the Reporting of OBservational studies in Epidemiology (STROBE) criteria (30).

## 3 Results

Between April 2019 and December 2021, an unselected cohort of 541 patients were treated with HoLEP for symptomatic benign prostate enlargement. A questionnaire asking for complications, IPSS, changes in sexual and continence function and patient global impression of improvement was sent to all patients with a response rate of 71.7% (388 patients). This questionnaire was sent at least 6 months after surgery with a mean follow-up-period of 14.9 ± 6.3 months.

 Table 2 shows the baseline characteristics of the study cohort.

**Table 2:** Baseline characteristics of the study cohort (*N* = 388).

Patients age [years]; M ± SD	72.1 ± 8.3
Preoperative IPSS; M ± SD	19.0 ± 7.7
Prostate volume [ml]; M ± SD	79.3 ± 41.7
Prostate volume ≥ 80 ml; *N* (%)	150 (38.7)
Preoperative indwelling catheter; *N* (%)	132 (34.0)
ASA-Status; *N* (%)	181 (33.5)
ASA 1	47 (12.1)
ASA 2	211 (54.4)
ASA 3	123 (31.7)
ASA 4	7 (1.8)
Anticoagulant medication; *N* (%)	143 (36.9)
Antiplatelet agents; *N* (%)	92 (23.7)
DOAC; *N* (%)	40 (10.3)
Coumarins; *N* (%)	9 (2.3)
Multiple anticoagulation; *N* (%)	2 (0.5)

IPSS: International Prostate Symptom Scale; TRUS: transrectaler ultrasound, ASA-Status: American Society of Anesthesiologists-Status; Antiplatelet agents: ASS or Clopidogrel; DOAC: Eliquis, Pradaxa, Xarelto, Lixiana.

The HoLEP procedure toke 63.0 ± 27.9 min and resected an adenoma with a median of 52.7 ± 35.3 g. The median length of postoperative stay was 2.51 ± 3.3 days with a transfusion rate of 0%.

Based on this questionnaire, a major complication rate of 1.8% within the follow-up period was reported in the cohort. This included postoperative bleeding, tamponade of the urinary bladder or need for bladder neck incision. There was no perioperative mortality. Within the questionnaire, 12.6% reported ambulant treated postoperative complications such as urinary tract infection, whereas 6.7% needed an additional treatment in the hospital by continuous irrigation due to bleeding or intravenous antibiotics.

We evaluated potential risk factors for postoperative complications, but could not identify any significant correlation between anticoagulation treatment (12.4% without and 15.9% with complication; *p* = 0.461), large prostate volume >80 ml (39.0% without and 43.2% with complication; *p* = 0.491), urinary retention with presence of an indwelling catheter at time of surgery (34.0% without and 34.1% with complication; *p* = 0.978) or ASA status ≥3 (33.0% without and 35.4% with complication; *p* = 0.688). None of these factors caused a higher rate of readmission or intervention under local or general anesthesia.

There was a significant change in the IPSS comparing before and after HoLEP (19.0 ± 7.2 vs. 6.2 ± 5.7; *p* < 0.001). Table 3 provides an overview of the postoperative functional outcomes. The occurrence of complications, the prostate volume, the postoperative IPSS, the IPSS change, changes in sexual function and the improvement of continence influenced the PGI-I significantly.

**Table 3:** Comparison of the patients answering the questionnaire regarding postoperative satisfaction.

	Total *N* = 388	Satisfied *N* = 305	Not satisfied *N* = 83	*p*-value
Age [years]; M ± SD	72.1 ± 8.3	72.1 ± 8.2	71.8 ± 8.8	0.799^a^
ASA > 3; *N* (%)	130 (33.5)	103 (33.8)	29 (34.9)	0.832^b^
Anticoagulation; *N* (%)	51 (13.1)	40 (13.1)	11 (12.9)	1.00^c^
Postoperative complications; *N* (%)	82 (21.1)	53 (17.4)	29 (34.9)	**<0**.**001**^b^
*Q*_max_ (preoperative); M ± SD	9.0 ± 5.7	8.5 ± 5.0	10.6 ± 7.4	0.258^a^
Prostate volume > 80 ml; *N* (%)	150 (38.7)	132 (43.3)	18 (21.2)	**<0**.**001**^c^
Weight of resected adenoma [g]; M ± SD	52.7 ± 35.3	55.2 ± 34.2	43.7 ± 38.1	**<0**.**001**^a^
IPSS (preoperative) *N* (%)	19.0 ± 7.7	18.2 ± 8.0	21. ± 6.1	0.005^a^
IPSS (postoperative); *N* (%)	6.2 ± 5.7	4.6 ± 3.7	13.0 ± 7.3	**<0**.**001**^a^
IPSS change (% from initial IPSS)	57.2 ± 64.7	63.5 ± 68.7	32.3 ± 36.0	**<0**.**001**^a^
Improving of urination; *N* (%)	331	296 (97.0)	35 (41.2)	**<0**.**001**^b^
Improving of sexual function; *N* (%)	44 (11.3)	42 (13.8)	2 (2.4)	**0**.**003**^c^
Deterioration of sexual function; *N* (%)*	124 (32.0)	81 (26.6)	43 (51.8)	**<0**.**001**^b^
Improving continence performance; *N* (%)	87 (22.4)	78 (25.6)	9 (10.8)	**0**.**004**^c^
Worse continence performance; *N* (%)	35 (9.0)	14 (4.6)	21 (25.3)	**<0**.**001**^c^

M, mean; SD, standard deviation.

Bold values represent statistical significance.

^a^ Mann–Whitney-*U*-Test.

^b^ Chi-Square-Test.

^c^ Fisher's Exact test.

**Figure 1:**
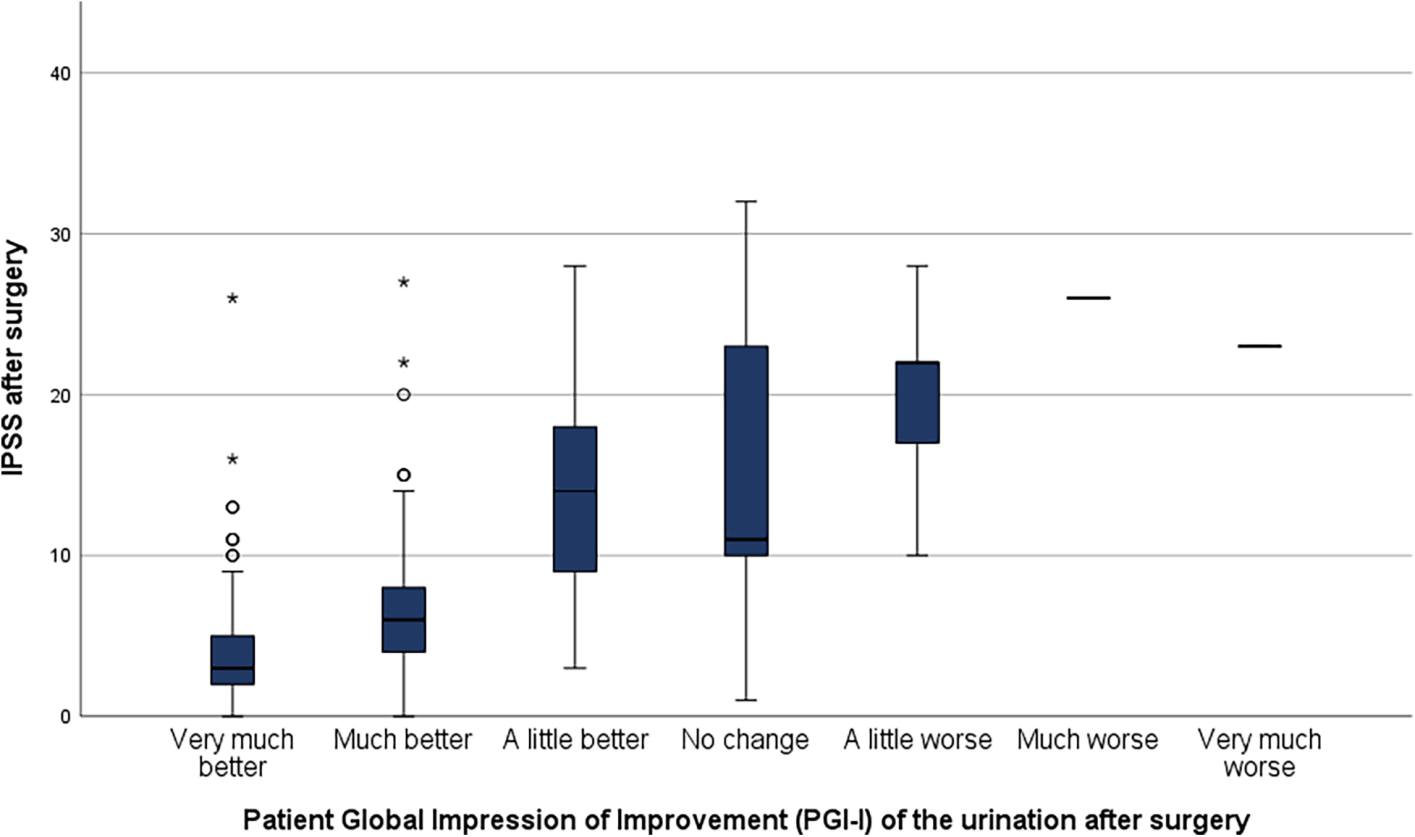
Correlation between patient global impression of improvement (PGI-I) and postoperative IPSS.

The used PGI-I showed a good correlation between reported satisfaction and the postoperative IPSS (Figure 1).

**Figure 2:**
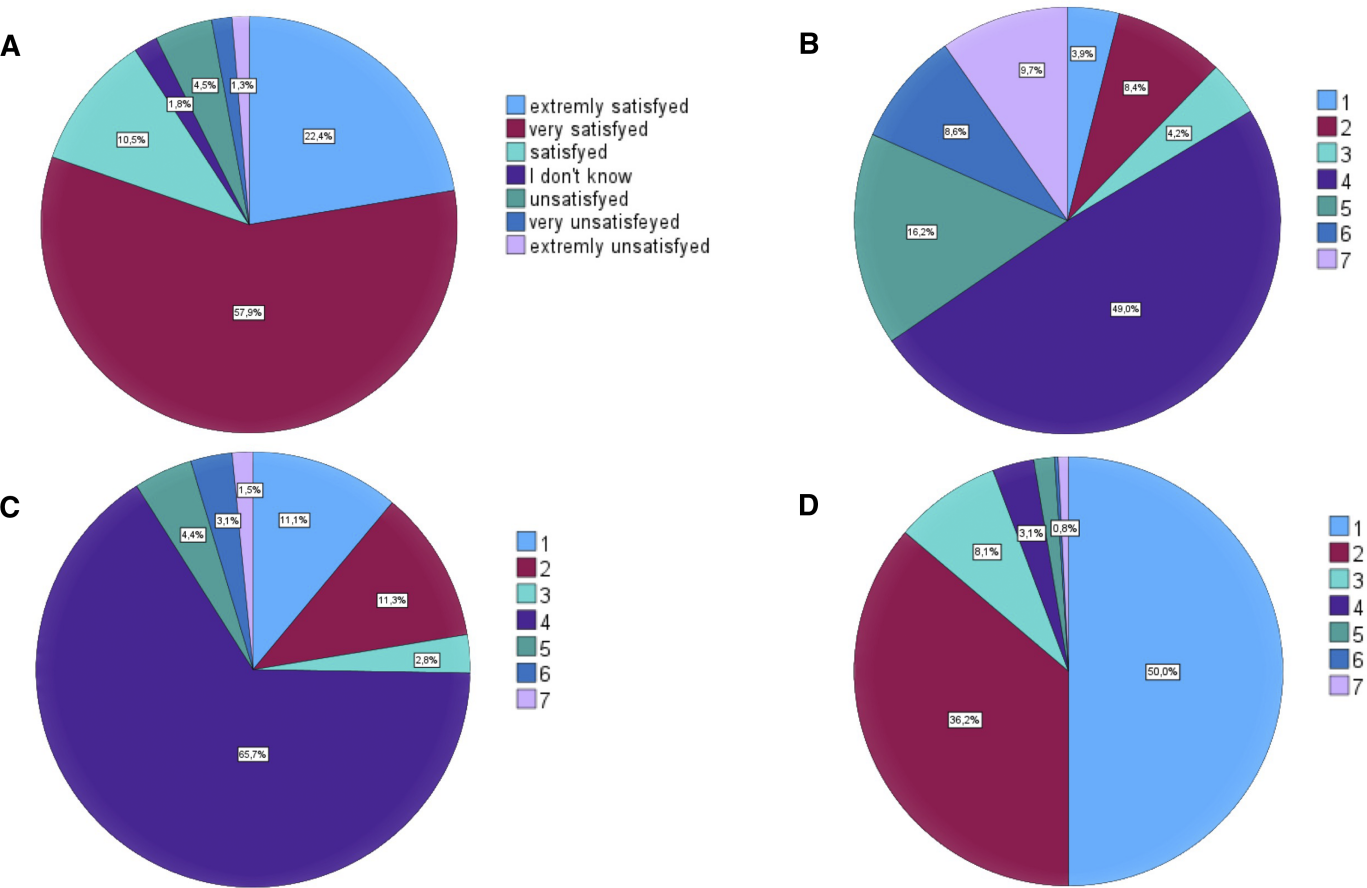
Patient reported outcome after HoLEP. (**A**) Satisfaction with result of surgery. (**B**) Changes in sexual function. (**C**) Changes in continence after surgery. (**D**) Changes in urination after surgery. In (**B**, **C**) and (**D**): 1: much better; 2: better; 3: a little better; 4: no change; 5: a little worse; 6: worse; 7: much worse.

While 19% of the patients reported a worsen sexual function after surgery, the majority of patients evaluated their postoperative outcome in a positive way (Figure 2A). This include 85% of patients rating the impact of their urinary symptoms as low. Ninety-four percent of patients reported a change towards a better urination after HoLEP (Figure 2B) and 91% are content with the surgical result. A total of 9% of the patients reporting their postoperative continence as worse in comparison to the preoperative situation (Figure 2C). This rate was significant lower in the group of satisfied patients (*p* < 0.001).

## 4 Discussion

This study underlines the effectiveness and safety of HoLEP using a high power Holmium Laser even in high-risk patients with anticoagulation and high prostate volume. The patients' satisfaction rate in this study cohort is high and easily measurable by a simple questionnaire (PGI-I) showing a good correlation to the postoperative IPSS and postoperative complications. This stresses the importance of PROM in the indication and quality control for surgical procedures.

The design of the questionnaire and the reported high correlation with the IPSS helps to establish an interview-based follow-up ensuring the measurement of patients' postoperative outcome, for example online or by phone call.

BPS and associated LUTS are benign diseases of aging men and are not deemed life-threatening, but they can have an apparently negative impact on patient´s QoL (6). Accordingly, measuring postoperative QoL is an important tool to evaluate the patients' satisfaction and outcome.

A good tool for the measurement of these results is provided by standardized questionnaires, which provide an insight into the personally perceived improvement or deterioration of certain parameters in the postoperative setting through targeted questions. The PROM collected in this way can individualize the optimization process in the surgical area and put the patient in an active role in this context. However, there is often a weak correlation to clinical parameters, which could not be observed in this study (31, 32). The used PGI-I correlated well with the postoperative IPSS, the occurrence of complications and the impact on the sexual function. Therefore, this simple and short questionnaire seems to be a valuable tool for a postoperative assessment after HoLEP.

Due to demographic change, the proportion of older patients will increase. It has been shown in several studies, that elderly patients benefit from a gentle procedure such as HoLEP (33, 34).

Older patients will also present with more comorbidities and also showing a higher rate of preoperative anticoagulation. Results from a German study indicated a significant higher risk of intra- and postoperative bleeding complications in HoLEP in patients with anticoagulation therapy (19). In this study, perioperative anticoagulation therapy was not associated with a reduced postoperative satisfaction. This is in accordance with the work of El Tayeb et al. (35) and Sun et al. (36). However, current limitations include: a lack of RCTs; limited data on short- and mid-term complications and bridging therapy; data presentation does not allow for separate interpretation of either antiplatelet and anticoagulation therapy (37).

Especially patients with a large prostate and a high weight of the resected tissue were satisfied with the procedure and their results. This might be due to the preoperative burden, these patients had. In larger glands obstruction as the most prevalent reason for LUTS is more likely.

While the preoperative IPSS did not differ significantly between both groups, the postoperative IPSS could be correlated with the postoperative satisfaction (Figure 1). The postoperative IPSS measured in this study is comparable to previously reported IPSS after HoLEP (1, 38). The comparability to other studies evaluating the postoperative IPSS after HoLEP is limited due to the evaluation at a single time-point without a longitudinal measurement.

Postoperative satisfaction rate after surgical interventions for BPS has been evaluated in several studies (1, 39–41) showing good results and are comparable to the 91% satisfaction rate reported in this study cohort.

Due to the continuous development of laser-technology, complication rates in HoLEP are decreasing. One example for this continuous evolution is the two-lobes-technique as used in this study. This is an optimization of the originally described 3-lobes-technique with a shorter length of surgery and lower incontinence rates (42). The optimal technique is still under debate and include 3-lobe, 2-lobe and en-bloc procedures as well as different ways of early dissection of the apical mucosa to reduce the tension to the sphincter region during the procedure (42, 43). Haemoglobin drop and operation time can be improved by pulse modulation of the laser (44). In this study, a major complication rate of 1.8% was reported by the patients. This is below the rate reported by Shoma et al. in 2023 (45). There was no higher complication rate in patients with reported risk factors such as anticoagulation (19) or high preoperative volume (46). But the rate of postoperative complications was significant higher in the group of patients with a reduced postoperative satisfaction, whereas this could be closely interlinked vice versa. This effect has been recently published in a population-based study by Berkowitz et al. (47).

### 4.1 Limitations

The major limitation of this study is its retrospective single-center design. Unfortunately, only limited longitudinal data are available and no preoperative quality of life is documented. Additionally, there is no internal or external control group reporting for example the outcome and quality of life after TUR-P in comparison to HoLEP, so an inter-group or pairwise comparison was not achievable. The survey period covers a very long period of time (6–27 month after surgery), which makes it difficult to evaluate especially the short- and long- time outcome. The age of the study cohort might influence some of the presented outcome parameters and make a long-term follow-up difficult.

Since we did not define any exclusion criteria preoperatively, parameters such as *Q*_max_ and IPSS could not be collected in patients with preoperative catheter.

All data regarding patient reported quality of life are limited by the subjective evaluation of every single patients, so a longitudinal evaluation comparing pre- and postoperative quality of life in the same patient would be a good basis for further research. Other techniques, such as the recently published telemonitoring might help to evaluate postoperative outcome, but till now, this technique is limited by the costs and patient's age restricting the use of new technologies.

## 5 Conclusion

The establishment of gentle, low-complication surgical procedures for symptomatic BPS for an unselected patient population is very important due to demographical changes. The HoLEP in two lobe technique was also confirmed in our study as a cohort-independent, safe procedure that leads to high patient satisfaction and a significant improvement in urination. In the study cohort, prostate volume, ASA score or preoperative anticoagulation did not show a significant influence on perioperative morbidity.

It is important in the indication of a surgical procedure for benign lesions, to include the patient reported outcome in the assessment and the follow-up. The data presented here show a good correlation between IPSS and PGI-I for postoperative patients' satisfaction. Further studies are needed to evaluate this in a longitudinal setting. In addition, a comparison to other treatment options, such as TUR-P or conservative treatments will help to define the best treatment for each patient.

In conclusion, this study showed a high procedure-specific satisfaction rate with a low complication rate, even for older patients with multiple comorbidities and the need of anticoagulation. In this context, the routine evaluation of patient-reported outcome can help choose the right treatment for each patient.

## Data Availability

The raw data supporting the conclusions of this article will be made available by the authors, without undue reservation.
